# Developing resources to facilitate culturally-sensitive service planning and delivery – doing research inclusively with people with learning disabilities

**DOI:** 10.1186/s40900-016-0031-1

**Published:** 2016-05-18

**Authors:** Gemma Unwin, Michael Larkin, John Rose, Biza Stenfert Kroese, Stephen Malcolm

**Affiliations:** 1 University of Birmingham, School of Psychology, Edgbaston, Birmingham B15 2TT UK; 2 St. Andrews Healthcare, Academic Unit, Northampton, UK; 3 Independent service user advisor, Dudley, UK

**Keywords:** Intellectual disabilities, Learning disabilities, Social support, Minority ethnic, Black, Asian, Inclusion, Partnership

## Abstract

**Plain English summary:**

(Please see www.Toolsfortalking.co.uk for an easy read summary of the project.)

The Tools for Talking are a set of resources that were developed through collaboration between Black, Asian and minority ethnic people with learning disabilities and researchers at the University of Birmingham. The resources were designed to be used by people with learning disabilities and service providers to facilitate culturally-sensitive communication and information sharing, service planning and delivery. They comprise illustrative videos and exploratory activities relating to five topics, namely, culture, activities, support from staff, important people, choices and independence. These topics emerged as important to people with learning disabilities during the ‘Access to Social Care-Learning Disabilities’ (ASC-LD) study which involved interviews with 32 adults with learning disabilities from Black, Asian and minority ethnic communities. The results of the ASC-LD study were used to develop a set of draft resources which were then co-developed through collaboration with people with learning disabilities and service providers. A ‘Partnership event’ was convened to involve stakeholders in the development of the resources. This paper describes the refinement of these materials by people with learning disabilities from Black, Asian and minority ethnic backgrounds in cooperation with a range of other stakeholders.

**Abstract:**

**Background**

Black, Asian and minority ethnic people with learning disabilities face inequities in health and social care provision. Lower levels of service uptake and satisfaction with services have been reported, however, this is largely based on the views of carers. The ‘Access to Social Care: Learning Disabilities (ASC-LD)’ study sought to explore the views and experiences of social support services among adults with learning disabilities from Black, Asian and minority ethnic communities. Interviews with 32 Black, Asian and minority ethnic adults with learning disabilities were conducted to explore participants’ cultural identities, their understanding and experience of ‘support’. The views and experiences expressed in the ASC-LD study were used in the ‘Tools for Talking project’ to develop a suite of resources designed to facilitate culturally-sensitive communication and information-sharing, service planning and delivery through improved mutual understanding between providers and users of services. This paper describes the Tools for Talking project which sought to co-develop the resources through a partnership event.

**Methods**

An inclusive approach was adopted to address issues that are important to people with learning disabilities, to represent their views and experiences, and to involve Black, Asian and minority ethnic people with learning disabilities in the research process. Partnerships were developed with provider organisations and service users who were invited to a ‘Partnership Event’. Collaborators at the partnership event were asked to comment on and evaluate draft resources which included a series of videos and activities to explore topics that emerged as important in the ASC-LD study. Their comments were collated and the tools developed as they suggested.

**Results**

Using the results from the ASC-LD study helped to ensure that the draft resources were relevant to service users, addressing topics that were important to them. The partnership event was an effective method to collaborate with a relatively large number of stakeholders. However, the event was resource intensive and required substantial planning to ensure active and meaningful participation. Considerations, such as inviting stakeholders, developing the programme and selecting a venue are discussed.

**Conclusions**

The partnership approach has led to the development of a set of five illustrative videos and accompanying activities that address issues that emerged from the collaborative process including: culture, activities, support from staff, important people, choices and independence. These resources are freely available at: www.Toolsfortalking.co.uk. They are designed to be used by users and providers of services, but may also be useful in other settings.

## Background

In England, information about satisfaction with statutory services is collected through the annual Adult Social Care Survey for England. The results of these surveys highlight lower quality of life, poorer access to services, and less satisfaction with social care services among people with Black, Asian and minority-ethnic heritage than those with White British backgrounds. Conversely, in the same survey, people with learning disabilities (also known as ‘intellectual disabilities’ and ‘intellectual and developmental disabilities’) tend to report high quality of life, and satisfaction with services. The surveys report these categories separately so do not allow researchers to tease out the experience of people with learning disabilities with Black, Asian and minority ethnic heritage. However, the findings are at odds with most research on services for people with learning disabilities and may indicate a ‘positive/acquiescence’ bias amongst the survey respondents. Existing research, primarily conducted with families (particularly South Asian British families) highlights inequities in service provision with carers reporting frustration and disappointment [1–6].

A small number of studies have accessed the views of Black, Asian and minority ethnic people with learning disabilities, especially in relation to social care provision. Black, Asian and minority ethnic participants with learning disabilities have reported social isolation, limited social networks, lack of leisure and recreational activities, unmet cultural needs and experiences of ‘double discrimination’ (in relation to disability and ethnicity), racism and stigma [8–10]. However, participants in these studies were broadly satisfied with the limited range of services received (for example, day services [8]) and mental health services [10]). One study explored ethnic and racial identity amongst adolescents and adults from South Asian communities [8]. The participants in this study expressed a strong and positive sense of their ethnic and cultural identities which was often constructed in terms of differences in features of cultural identity, both with majority populations and between South Asian cultures.

The Access to Social Care-Learning Disabilities (ASC-LD) study sought to explore the views and experiences of social care services among Black, Asian and Minority Ethnic adults with learning disabilities [7]. Thirty-two adults with learning disabilities from the most prominent minority ethnic communities in the West Midlands region of the UK, namely people with Pakistani, Bangladeshi, Indian, Caribbean, and African heritage were interviewed. The researchers employed various techniques to assist participants in articulating their experiences and ideas in the interviews. The authors identified a series of broad topics that were important to the participants including culture and identity, independence, relational networks, current and desired activities, and good support. The ‘Tools for Talking’ project outlined here sought to develop the findings of the ASC-LD study, through an inclusive and participatory process, to create a set of resources to facilitate culturally-sensitive service planning and delivery.

The importance of good support emerged as a clear message from the participants in the ASC-LD study: good support was conceptualised as good relationships with support workers and the participants were generally positive about these relationships. Views and experiences around independence varied, some participants expressed tensions and some felt ambivalence towards what they and others perceived to be independence. This was most pertinent in relation to participants living in their own home and in terms of reductions in support. Daily activities were also very important:he participants talked about a wide range of activities that were important to them for lots of different reasons. For example, going to the gym and on bike rides were important to keep fit and healthy; activities were also important to socialise, meet other people and have fun.

There are numerous guidance and policy documents published in the UK pertaining to social and health care provision to people with learning disabilities from Black, Asian and minority ethnic communities (e.g. [11–17]). The reports highlight the current lack of appropriate service provision for minority-ethnic people with learning disabilities and their families resulting in their under-representation in services. Issues around double discrimination, marginalisation, institutional racism, dissatisfaction and disillusionment are considered. The reports recommend that services invest more effort to reach out to communities and involve them in planning and delivering services, to take a personalised approach to service provision, and to provide services that meet the cultural and holistic needs of service users and their families. One of these reports utilised a participatory research model with young people with learning disabilities from ‘South Asian’ communities recruited as co-researchers to investigate leisure interests and needs [17]. The co-researchers conducted focus group discussions with 24 South Asian young people with learning disabilities. They reported barriers to access, including a lack of support and transport. Parents often provided support and access to transport, however, this created problems when parents had competing commitments. They suggested that increased support around ‘taking medication, combating bullying, doing things at the right time, handling money and writing’ would facilitate access to leisure.

The main aim of this paper is to describe and review a partnership event which was aimed at refining and developing a set of resources that could help implement current best practice recommendations. The Tools for Talking project sought to address some of the key concerns identified in the narratives of the participants in the ASC-LD study [7, 18] by developing a set of resources that would facilitate culturally-sensitive communication and information sharing, service planning and delivery through improved mutual understanding. The aim was to hear the voices of people with learning disabilities with Black, Asian and minority ethnic heritage, to understand their point of view, to feed these experiences back to people with ID, and to collaboratively develop the resources. Both the ASC-LD study and the Tools for Talking project were underpinned by a commitment to empowering people with learning disabilities to tell their stories, to highlight and reduce barriers and improve the life chances of people with learning disabilities with Black, Asian and minority ethnic heritage through developing an evidence base which could inform service development.

Consultation with people with learning disabilities was integral throughout the ASC-LD study [7, 18] and Tools for Talking project. Two people with learning disabilities provided ongoing input throughout the project as members of the Steering Committees. Partnerships were also established with third sector organisations and an advocacy group. The ASC-LD study sought to identify issues pertinent to people with learning disabilities with Black, Asian and minority ethnic heritage. The participants in the ASC-LD study therefore ‘set the agenda’ for the Tools for Talking project as the participant-led discussions in the ASC-LD study were used to identify key issues in social care service use.

## Methods

### The development of and production of the draft resources

The interview transcripts from the ASC-LD study were used to develop five topic areas. The key messages were distilled from the interviews whilst representing a broad range of views and experiences, and remaining close to the words of the participants, using direct quotes where possible. We sought to represent the key messages from the interviews through the use of personal, first-person narratives. The five topics identified in the ASC-LD study were developed into draft resources (the Tools for Talking) which were intended to be used by service users in association with service providers.

**Fig. 1:**
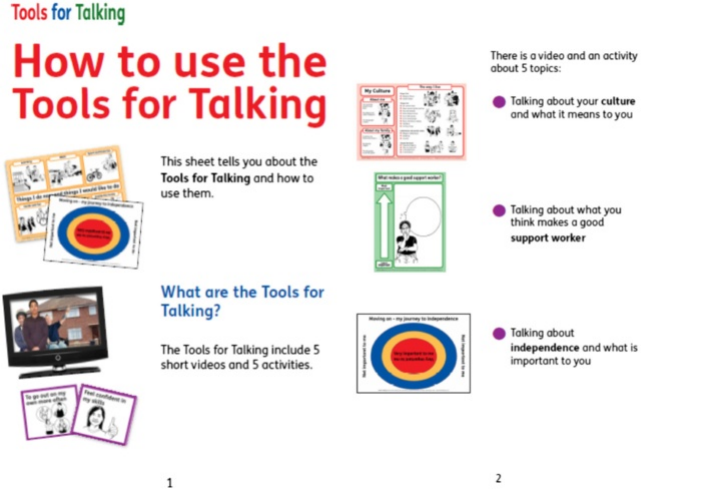
Example of the instructions for service users

Five videos, which took the format of digital stories (audio set to static images), and five accompanying activities were developed. The activities were designed to be simple, easy to use, and engaging to facilitate communication between service user and provider. The activities comprise a poster to prompt and structure discussion. Further resources are provided such as worksheets, cards and stickers. Two of the activities involve a card sort to rate the relative importance of items (‘what makes a good support worker?’ and ‘moving on–my journey to independence’). A further activity uses thermometer stickers for service users to rate the relative salience of features of their cultural identity. Another activity uses cards as prompts to help service users think about the range of activities that may be available (‘things I do now and things I would like to do’). The final activity (‘important people in my life and my relationships with them’) maps out relational networks and facilitates people to explore the meaning attached to relationships through the application of stickers. Two sets of instructions are provided for each activity–one for support workers and an easy read set of instructions for service users. The activities were designed to provide a framework for people with learning disabilities to tell the people around them about their preferences the meanings they attach to these preferences. The videos were designed to illustrate each topic, we therefore recommend that users watch the video first, then complete the activity (Fig. 1).

### The partnership event

The draft resources were taken to a Partnership Event to further develop them. The Partnership Event was designed to get specific feedback on the materials and harness the expertise of the anticipated end-users of the Tools for Talking, namely, service users and service providers (including support workers and managers of provider organisations). The draft Tools for Talking were showcased at the event and attendees were asked to provide feedback and suggestions for their development. This provided structure and focus for the event, but we did not constrain comment to feedback on the drafts, rather we encouraged elaboration through discussion and were open to feedback not directly related to the resources. Extensive planning was undertaken and was informed by discussions with the Steering Group, a representative from an advocacy organisation and learning disability health professionals (as member of the project team).

A number of practical considerations were identified for consideration in the planning phase. When deciding on the venue for the event it was important to consider access, including disabled access, within the building and parking space for those arriving by car. We also discussed whether the event should be located at University conference facilities or a more neutral venue. Service user advisors suggested that participants may enjoy the opportunity to visit the campus, therefore, the event was held at University conference facilities. Furthermore, we arranged for a quiet room, adjacent to the main room to allow participants some space should they need it.

We considered how participants would travel to and from the event. It was anticipated that some would travel independently on public transport, however, some may require personal taxis. We therefore budgeted for each participant to receive fixed travel expenses. This was offered without the need for receipts as we felt this could be problematic for some individuals. We also offered a gift voucher to all of the attendees, as a token to say thank you for their time. This was offered as a gift voucher to avoid participants having to complete fee claim forms. Lunch was provided but it was restricted to vegetarian only to respect the dietary requirements of some attendees.

#### Invites

Originally, we considered inviting representatives from a wide range of stakeholder groups, including commissioners, health professionals and local authority representatives. However, after discussion, we narrowed the target group for the event to service users (adults with learning disabilities and Black, Asian and minority ethnic heritage) and service providers (direct care workers and managers of service provider organisations) to allow us to co-develop the tools with the anticipated end users. The majority of the service users and providers had already been involved in various aspects of the ASC-LD project and we used snowballing to identify others to invite.

Care was taken to develop an attractive, easy read, postal invitation for service users. A simple reply slip and pre-paid postage envelope were also supplied. Where appropriate, follow-up phone calls were made to discuss travel arrangements and support needs. All service users were offered the option of inviting someone to accompany them to the event.

#### Participants

The event was attended by 43 people including 25 service users, 11 support workers/relatives, and 7 service managers. Eleven project group members/facilitators were also present. There was a good response rate to the invitations sent to service users. This may be due to previous involvement in the project, relationships developed with the project team and follow-up phone calls. Some participants attended independently, others were accompanied by family members and support workers.

#### The programme for the event

**Fig. 2:**
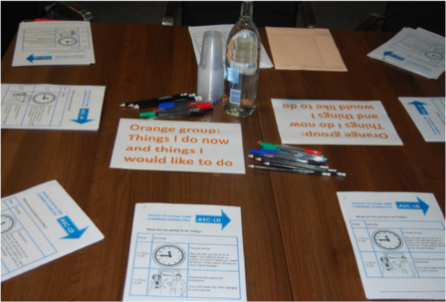
The partnership event

On arrival, participants were assigned to a colour-coded group. Each group was based around a single table and participants were allocated to one of the five topics. Care was taken to try to allocate participants to groups where they would be with someone they knew to provide a sense of familiarity (Fig. 2).

The event lasted 3 h and 15 min, including lunch. It was decided to keep sessions relatively short and interactive to maintain attention and engagement. The event opened with a short presentation to provide an overview of the project and set the aims for the day. The videos were then played, each followed by a voting session for the service users. The voting session aimed to gather initial feedback from participants without the expectation that they would have to articulate their views. Three statements were posed after each video to obtain feedback on whether the content of the videos was understandable (‘the words and language were clear’), how relevant the content felt to participants (‘I think these things too’), and whether the content resonated with participants (‘it was good to hear what other people think’). Each statement was read out in turn with explanations provided for how to respond. Participants were asked to vote by holding up a piece of coloured paper. There were three choices: red (disagree), yellow (neutral) and green (agree). Photographs were taken to capture the responses so that these could be counted at a later date. A short discussion session followed the voting. Facilitators asked the groups to reflect on what they saw, to elaborate on how relevant the videos felt, to consider the style and format of the videos and suggestions for improvements.

**Fig. 3:**
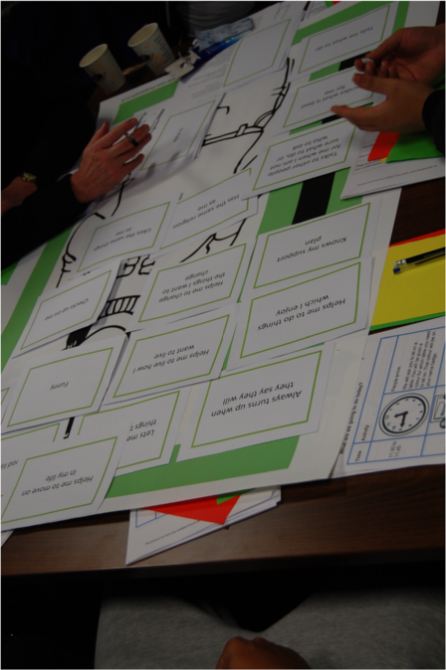
The activities at the Partnership Event

In a second part of the event participants were asked to participate in one of the five activities. Participants watched the appropriate video again to orient them to their activity. Groups were encouraged to discuss and collate feedback on the process. Service providers facilitated the activities and supported service users in discussion (Fig. 3).

## Results and discussion

### Feedback on the resources

The response to the videos in the event was generally very positive with a majority of green cards displayed. The ‘Important people in my life and my relationships with them’ video received the most yellow cards (*n* = 11) indicating lower relevance for this video. Feedback from the discussion of the videos highlighted that the speaking pace in the videos was too fast and there was a preference for photographs over line drawings. For the final resources, the audio was re-recorded with people with learning disabilities and Black, Asian and minority ethnic heritage reading the scripts, at a slower pace. The line drawings were also replaced with photographs.

The participants enjoyed hearing about what other people thought and particularly resonated with the video outlining independence. They felt it captured some of the ambivalence relating to how service users feel about independence. However, they felt it was too focussed on independence meaning living in your own home. This video were therefore re-worked to include two narratives, one of a service user working towards increased independence, but remaining in a group community home, and another discussing their previous move from a group home to supported living accommodation.

**Fig. 4:**
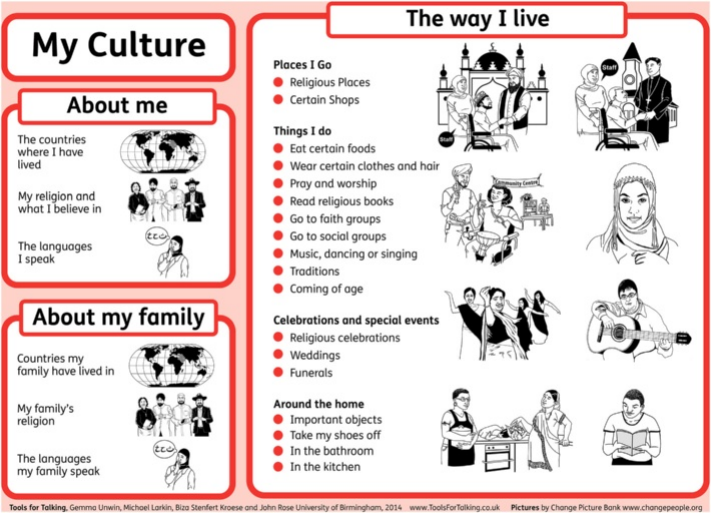
The Culturegram

One activity is based around a ‘culturegram’ which is a creative tool to help service users explore aspects of their culture. We received suggestions for refinements to the culturegram such as additional dimensions (e.g. practices within the home, festivals and celebrations), examples within these dimensions and the inclusion of a system to rate the relative importance of various aspects of a person’s cultural identity. It proved challenging to strike a balance between providing examples and prompts within the resources without being prescriptive, perpetuating stereotypes and attempting to reflect the diversity in cultural identity among our intended end users. This was most noticeably borne out when selecting images for the culturegram whereby space was limited on the poster and worksheet. Instructions and discussion prompts cards are therefore provided for service providers to facilitate discussion around this complex topic (Fig. 4).

Another activity seeks to identify features of good support in relation to support workers. This activity was well received, however, it was felt that there were too many cards to arrange in order of importance. Group members also felt the cards should reflect a more balanced range of attributes to make the activity more interesting as most of the cards were rated as important for good support. A more balanced set of cards was subsequently developed, however, as the aim of the activity was to address ‘good support’ (as emphasised in the ASC-LD study) rather than poor support, the cards were still aligned with that, but with more ambiguous cards that could stimulate discussion and make the activity more interesting.

Another activity involves service users organising various activities in order of importance when thinking about independence. The group felt that the poster for this activity included too many levels of importance, represented as ‘rings’ on the poster, and suggested that three levels rather than five would be sufficient. They also provided suggestions for further example cards. A different activity was designed to facilitate service users in thinking about their daily activities. Feedback indicated that this activity needed further development to help users move from mapping out what they currently do to thinking of things they would like to do. Issues around service planning and development were also highlighted which led to the development of a planning tool to help service users and providers identify and discuss potential barriers to participation in activities.

### Feedback on the event

Feedback on the event was sought through easy-read feedback forms. The feedback was very positive; everyone enjoyed the day and the activities. Only one person felt that we did not say things in a way that they understood. Comments on the feedback forms included:

“Yes, I agree with things being said and presented at the start and the project is a good idea.”

“Good project, we should do this more often.”

“The day was very enjoyable and met different people with many interesting views.”

“Good to see so many people involved, enjoying it and being listened to.”

“I enjoyed the project that we did and all the different opinions that people said, it got me thinking.”

“Everyone had time to say how they felt.”

### Reflections on the event

The mood was really positive during the event and everyone engaged with the activities. The programme seemed appropriate and well-paced. The event proved to be a useful method to involve a large group of people with learning disabilities in project development. However, it was resource intensive and time consuming. Planning the event took several months and a large team of facilitators were required on the day to help with the activities, to facilitate discussion and to record feedback. Payment of participants’ travel expenses and for their time required a sizable budget. Preferably, participants would have been paid a wage for their involvement–based on their commitment of over three hours of their time–but budgetary and administrative constraints restricted payment in this instance.

We expected a proportion of confirmed participants not to attend on the day, however, almost all attended. This resulted in groups that were too large and a rather cramped and a noisy room. A larger room would have been better with more space between the tables to reduce noise between groups.

There was a difficult balance to strike between keeping the event relatively short, and achieving our aims. During the voting section of the day, we felt conscious of the pressure to maintain momentum, and hold everyone’s attention, but we were aware that this might also have limited participants’ opportunities to fully consider their answers. The pace and level of activity might have led to some acquiescent responses, biased towards the positive. We had considered counter-balancing the questions but felt this would be too confusing. Perhaps allowing more time and posing more concrete questions relating to pace of the videos, use of images, and content would have yielded more balanced feedback.

People were interested in taking the work further and attending future events, however there was no resource for this. Future research should consider how to build and maintain ongoing relationships with service users and service providers to establish networks for inclusive research. Inclusive research tends to omit those with more severe learning disabilities [19]. This is a limitation of our study because only those with mild/moderate learning disabilities were involved. The Partnership Event approach may not lend itself to the inclusion of those with more complex needs. A more individualised approach may be more suitable for those with limited communication. For example, the Tools for Talking could be piloted with people with limited verbal communication and, rather than seeking explicit feedback, we could observe and feedback how the service user engaged with and responded to the activities.

Previous inclusive research has identified priority areas for further research, including, access to healthcare, communication with healthcare professionals, relationships (including friendships and parents with ID), employment, inclusion (including being able to do things in the community), discrimination, support, and independence [20–22]. These issues overlap with the topics identified in the present project in relation to support, independence, relationships, and activities which indicates transferability of our results. The differences may be anticipated by our focus on support services rather than health services. Moreover, the addition of cultural identity is a strength of our study as it has been previously overlooked when assessing satisfaction with services [23]. The Tools for Talking may therefore be useful for further research into how we can improve services. The culturegram may be especially useful in research and clinical/service settings to aid the assessment of cultural identity, amongst those from minority-ethnic as well as majority communities.

There is currently little conceptual clarity to determine the features of inclusive research and to guide its conduct, but it is driven by a commitment to a ‘respectful relationship and regard for the perspectives of people with [learning] disability’ [19]. Authors suggest that inclusive research should address issues that matter to people, leading to improved lives, should represent the views and experiences of people with learning disabilities, and should treat people with learning disabilities with respect [24]. The present project used the results of the ASC-LD study to ‘set the agenda’ to ensure that we represented the views and experiences of people with learning disabilities with Black, Asian and minority ethnic heritage Furthermore, authors argue for a flexible and expansive concept of how to do inclusive research and how to do it well and suggest that researchers should think more about how to do research inclusively rather than how to do ‘inclusive research’ [25]. The Tools for Talking project took this latter approach to develop a method to include a relatively large group of service user and providers in the development of the resources.

Bigby and colleagues review the literature on inclusive research with people with learning disabilities in an attempt to provide conceptual clarity. They suggest that inclusive research comprises three approaches, namely ‘advisory’, ‘leading and controlling’ (people led), and ‘collaborative group’ (where people with LD and researchers have equal partnerships with expertise in different areas). The authors argue that each has advantages and challenges and the choice of approach should be informed by the nature of the project, including the aims, resources and topic of investigation. We selected to use an ‘advisory’ process to harness the expertise of service users and providers in the development of the resources. One strategy was to use a Partnership Event which was felt to meet the aims of the study. We wanted to produce resources that were widely applicable and representative and therefore wanted to involve a relatively large number of people. This is a relatively novel approach as most studies express involvement through involvement of users in advisory committees, as conduits for recruiting other service users, to analyse data, as supporters or advocates of other service users, or in dissemination [26].

We used the same sampling methods for our partnership event as was used in the ASC-LD study and sought representation from the more prevalent ethnic minorities in the West Midlands region. We therefore did not consult with representatives from all minority ethnic communities, such as newer migrants. However we sought to create resources that were flexible and applicable across all adults with learning disabilities. Indeed, feedback through dissemination activities indicates that the resources are equally relevant for majority groups. The issues which emerged in the ASC-LD study were largely applicable to all adults with learning disabilities and are not unique to those with minority ethnic heritage. Many of the concerns relate to basic, fundamental aspects of good support and emphasise that providing services to people with Black, Asian and minority ethnic heritage should not be considered as a separate endeavour, rather, it is accounted for by provision of personalised, individualised services.

## Conclusions

The Tools for Talking are to help service users think about what is important to them, to help them communicate their preferences and the meaning they attach to these preferences, and to help service providers understand service user’s wants, needs and desires. They are freely available at: www.Toolsfortalking.co.uk.

Partnership Events are an engaging and suitable way to include relatively large numbers of service users in research. However, they are resource intensive and require careful planning.

This paper forms part of ongoing dissemination of the project, which has already included a launch event to which social and third sector service providers were invited, and a series of presentations to social care providers. We also produced and distributed a large number of printed manuals containing the activities and DVDs of the videos. The Tools for Talking have been positively received and social care providers fed back that they would incorporate them into their practice. However, future research should seek to evaluate their use.
